# Facile synthesis and optical properties of polymer-laced ZnO-Au hybrid nanoparticles

**DOI:** 10.1186/1556-276X-9-109

**Published:** 2014-03-07

**Authors:** XianHong Wang, XiaoYan Zhang, WenZheng Cheng, HongQin Shao, Xiao Liu, XueMei Li, HongLing Liu, JunHua Wu

**Affiliations:** 1 Key Lab of Polyoxometalate Chemistry of Henan Province, Institute of Molecular and Crystal Engineering, School of Chemistry and Chemical Engineering, Henan University, Kaifeng 475001, China; 2 Pioneer Research Center for Biomedical Nanocrystals, Korea University, Seoul 136-713, South Korea

**Keywords:** Nanoemulsion, ZnO-Au nanoparticles, Polymer, Optical properties, 78.67.Bf, 36.20.-r, 68.05.Gh

## Abstract

Bi-phase dispersible ZnO-Au hybrid nanoparticles were synthesized via one-pot non-aqueous nanoemulsion using the triblock copolymer poly(ethylene glycol)-*block*-poly(propylene glycol)-*block*-poly(ethylene glycol) as the surfactant. The characterization shows that the polymer-laced ZnO-Au nanoparticles are monosized and of high crystallinity and demonstrate excellent dispersibility and optical performance in both organic and aqueous medium, revealing the effects of quantum confinement and medium. The findings show two well-behaved absorption bands locating at approximately 360 nm from ZnO and between 520 and 550 nm from the surface plasmon resonance of the nanosized Au and multiple visible fingerprint photoluminescent emissions. Consequently, the wide optical absorbance and fluorescent activity in different solvents could be promising for biosensing, photocatalysis, photodegradation, and optoelectronic devices.

## Background

Multi-constituent nanomaterials with diverse compositions and tailorable morphology exhibit multiple functionalities and novel properties, showing prospective potentials in biological detection and sensing, drug delivery, hyperthermia, cell separation, magnetic data storage, strong catalysis, photoelectric conversion, and many other areas [1-3]. Syntheses of such nanoparticles and investigating their properties are hence of general interest. On one hand, gold nanoparticles as a typical noble metal product, because of their chemical stability, original biocompatibility, and prevailing effects of surface plasmon resonance in the visible region, offer excellent, versatile opportunities in immunoassay, biosensing, and optimal catalysis [4-8]. On the other hand, ZnO nanoparticles with a wide energy bandgap are an excellent, well-studied semiconductor, accompanied by shifting of the intrinsic band due to quantum confinement [3,9-11]. Strong, tunable absorption and emission bands revealed in ZnO nanostructure, characterized by the particle size and the surrounding medium, have found uses in biosensing technology, electronics, photoelectronics, catalysis, and chemical degradation.

By nanoengineering these two materials into a single entity, the ensuing nanostructure would not only exercise the unique properties of gold and the semiconductor, but also generate novel collective phenomena based on the interaction between Au and ZnO [12-15]. Such a structural nanoassembly can have the extra advantages of biocompatibility and low toxicity and afford an easy, effective contact between biological tissue and the nanoparticles, anticipated to be benign for biological detection, photocatalysis, and dye-sensitized solar cells. Ranking in a variety of interesting structural forms, the synthesis of ZnO-Au nanoparticles has been performed for various purposes [16-21]. In addition, the natural coating of nanoparticle surfaces by an ultrathin film of biocompatible molecules is highly desirable for future biomedical applications, especially if done *in situ* during the synthesis process of the nanoparticles [3,17]. We here report the preparation of ZnO-Au hybrid nanoparticles by one-pot non-aqueous nanoemulsion with the triblock copolymer poly(ethylene glycol)-*block*-poly(propylene glycol)-*block*-poly(ethylene glycol) (PEO-PPO-PEO) as the surfactant. The copolymer has proved many distinctive merits, such as aqueous solubility, biocompatibility, non-charging, and non-toxicity, and is often used in a number of fields [22-26]. In nanoemulsion processes, the PEO-PPO-PEO molecules principally participate in the reactions as a surfactant, playing a role in stabilizing the nanoparticles formed and even acting as a reducing agent, as attested in our reports on long-term stable, highly crystalline, monosized Fe_3_O_4_/Ca_3_(PO_4_)_2_, Fe_3_O_4_/ZnO, Fe_3_O_4_/Au, and FeAu nanoparticles [3,8,27,28]. In this work, the ZnO-Au nanoparticles prepared without a secondary surface modification were bi-phase dispersible. The characterization shows that such polymer-laced ZnO-Au nanoparticles are monosized and of high crystallinity and possess excellent dispersibility and optical performance in both organic and aqueous medium.

## Methods

Bi-phase dispersible polymer-laced ZnO-Au nanoparticles were prepared by one-pot non-aqueous nanoemulsion, using gold acetate and zinc acetylacetonate as the precursors, the triblock copolymer PEO-PPO-PEO as the surfactant, and 1,2-hexadecanediol as the reduction agent. Typically, 0.25 mmol of gold acetate, 0.25 mmol of zinc acetylacetonate, 0.1358 mmol of PEO-PPO-PEO, and 2.5 mmol of 1,2-hexadecanediol were mingled in 10 ml octyl ether in a 250-ml flask under vigorous stirring. The reaction mixture was first slowly heated to 125°C within 2 h and homogenized at this temperature for 1 h under vigorous stirring, then rapidly heated to 280°C within 15 min and refluxed at the temperature for 1 h. After cooling down to room temperature, ethanol was added to the reacted solution to precipitate the PEO-PPO-PEO-laced ZnO-Au nanoparticles by centrifugation. The precipitated product was washed with ethanol/hexane (2:1) several times. The resultant nanoparticles prepared in such a process can be re-dispersed in hexane, ethanol, and distilled water directly, without a secondary surface modification which is usually required [17]. For comparison, Au and ZnO nanoparticles were prepared similarly using only gold acetate or zinc acetylacetonate as the precursor.

The morphology of the ZnO-Au nanoparticles was analyzed by transmission electron microscopy (TEM, JEM-100CX), whereas the structure was characterized by X-ray diffractometry (XRD, X'Pert Pro, PANalytical B.V., Almelo, The Netherlands; *λ* = 1.54056 Å) using Cu K_α_ radiation. An Avatar 360 Fourier transform infrared spectroscopy (FTIR) spectrometer (Nicolet Company, Madison, WI, USA) was applied to perform the Fourier transform infrared spectroscopy investigation. In the FTIR studies, the washed ZnO-Au nanoparticles and the pure PEO-PPO-PEO polymer employed in the preparation were crushed with a pestle in an agate mortar, the individually crushed material was mixed with potassium bromide (IR spectroscopy grade) (Merck, Darmstadt, Germany) in about 1:100 proportion. The mixture was then compressed into a 2-mm semitransparent disk by applying a force of 10 t for 2 min. The FTIR spectra were recorded at the wavelength range of 400 to 4,000 cm^-1^. Moreover, the optical properties of the ZnO-Au nanoparticles separately dispersed in hexane, ethanol, and water, together with the Au and ZnO nanoparticles in hexane, were characterized by an UV-visible spectrophotometer (UV-vis near IR spectrophotometer, Hitachi U4100; Hitachi, Shanghai, China) and a photoluminescence (PL) spectrophotometer (Hitachi F7000, Japan).

## Results and discussion

**Figure 1:**
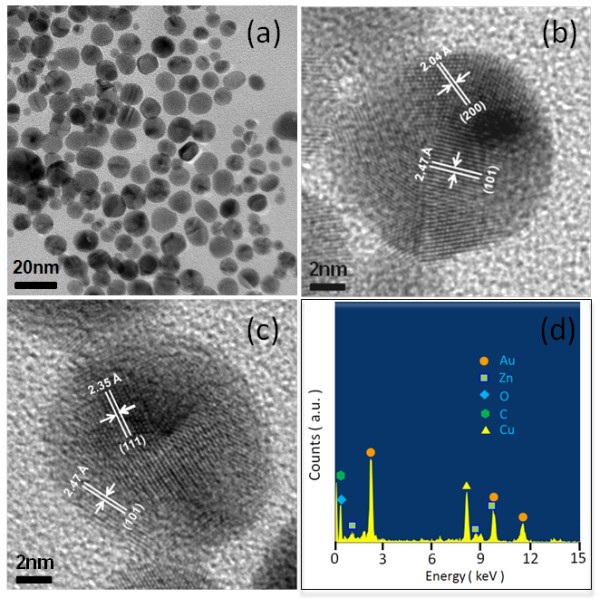
**TEM analysis of the polymer-laced ZnO-Au hybrid nanoparticles. (a)** Bright-field image. **(b, c)** HRTEM of individual nanoparticles. **(d)** Point-detection EDX analysis of the composition.

The morphology and particle size of the prepared ZnO-Au hybrid nanoparticles are shown in Figure 1a. Apparently, the nanoparticles are highly crystalline, virtually uniform, and spherical in shape. The particle size histogram from the size counting of the nanoparticles acquired from a series of TEM images shows a tight size distribution which is described quite satisfactorily by a Gaussian function and gives an average particle size of approximately 9.9 nm in diameter and a standard deviation of 1.1 nm. The HRTEM images in Figure 1b,c show two individual examples of the ZnO-Au nanoparticles in the highly crystalline state. Indicated in Figure 1b are the projected (200) plane for Au and the (101) plane for ZnO and in Figure 1c the (111) plane for Au and the (101) plane for ZnO, individually. The observation directly illustrates the coexistence of Au and Zn in the same nanocrystals, with the incorporation of both cubic Au nanocrystallites and ZnO hexagonal wurtzite nanostructure as further corroborated in the following XRD examination. The phenomena imply that Au does not intermix strongly with ZnO, but light doping and/or partial alloying is still possible. Figure 1d shows a typical TEM-EDX point-detection instance for the composition, clearly exposing the simultaneous presence of both zinc and gold elements.

**Figure 2:**
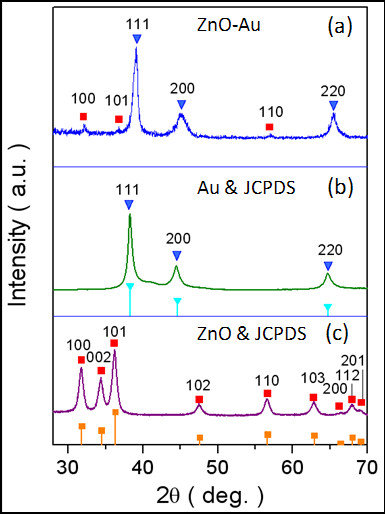
**X-ray diffraction patterns of the various nanoparticles. (a)** ZnO-Au. **(b)** Au (bar diagram for the JCPDS of bulk Au). **(c)** ZnO (bar diagram for the JCPDS of bulk ZnO). Au in inverted triangles and ZnO in squares.

The nanoparticles were further investigated by the X-ray crystal structural analysis. As shown in Figure 2a, the diffraction peaks of the ZnO-Au nanoparticles may be indexed to two sets, one in the inverted triangles corresponding to the Au positions of the (111), (200), and (220) planes, and the other in the squares corresponding to the ZnO positions of the (100), (101), and (110) planes. The findings are substantiated by the diffraction pattern of Figure 1b recorded for the Au nanoparticles prepared from gold acetate (JCPDS no. 01-1172) and that of Figure 1c obtained for ZnO nanoparticles synthesized from zinc acetylacetonate (JCPDS no. 36-1451). As regards to the result of the hybrid nanoparticles, the dominant Au intensities may be attributed to the much stronger scattering power of the material than that of ZnO [29]. The observation of the ZnO (100) family of planes and the absence of the ZnO (002) family of planes clearly supports the nanostructuring of ZnO and Au in a single motif. In addition, the average particle size of the ZnO-Au nanoparticles is estimated to be approximately 8.9 nm by the Scherrer equation based on the full width at half maximum (FWHM), comparable to that from the statistical size counting of the TEM analysis above, supposing that the broadening of the peaks in the XRD pattern is predominantly due to the finite size of the nanoparticles [30].

**Figure 3:**
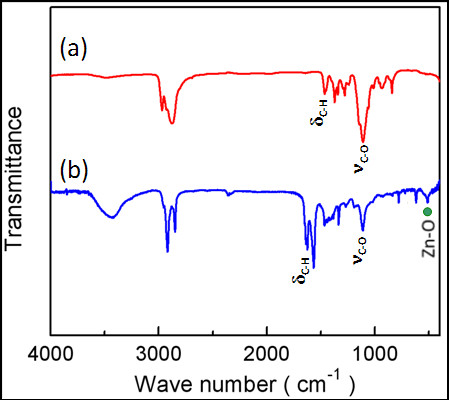
FTIR spectra of (a) the pure PEO-PPO-PEO polymer and (b) the PEO-PPO-PEO-laced ZnO-Au hybrid nanoparticles.

The determination of existence of the PEO-PPO-PEO macromolecules on the surface of the ZnO-Au nanoparticles was undertaken by comparatively assessing the FTIR spectra of the pure PEO-PPO-PEO polymer and the polymer-laced ZnO-Au nanoparticles after purification [22-27]. In Figure 3a, the pure PEO-PPO-PEO polymer molecules display one strong characteristic band at the position of approximately 1,108.92 cm^-1^ due to the C-O-C stretching vibration of the ether bonding which usually ranges between 1,250 and 1,000 cm^-1^, and one sharp characteristic band at the position of approximately 1,465.71 cm^-1^ due to the C-H bending vibration. As given in Figure 3b, these characteristic vibration and bending features reappear in the FTIR spectrum of the PEO-PPO-PEO-capped ZnO-Au nanoparticles, but blueshifting to the positions of approximately 1,115.63 cm^-1^ for the C-O-C stretching vibration and approximately 1,625.26 cm^-1^ for the C-H bending vibration [27,28], respectively. Evidently, the vibration and bending shapes and absorption intensities vary between the pure PEO-PPO-PEO molecules and the PEO-PPO-PEO-covered ZnO-Au nanoparticles. Both blue-shifting and shape change in the C-O-C stretching and C-H bending modes may be attributed to the interactive coordination of the oxygen atoms in the PEO-PPO-PEO main chains to the Au and Zn atoms in the hybrid nanostructure [27,28,31]. Consequently, the observation provides strong evidence that the PEO-PPO-PEO molecules are coated onto the surface of the ZnO-Au nanoparticles, as the redundant PEO-PPO-PEO molecules were removed by the washing procedure. As a result of such PEO-PPO-PEO lacing, these PEO-PPO-PEO-ZnO-Au nanoparticles turn out to be both hydrophobic and hydrophilic, which are entitled a bi-phase dispersible property intended for an easy transport of the nanoparticles between non-polar and polar solvents without further surface modification, as demonstrated in the study on the optical properties of the nanoparticles in the subsequent sections [17].

**Figure 4:**
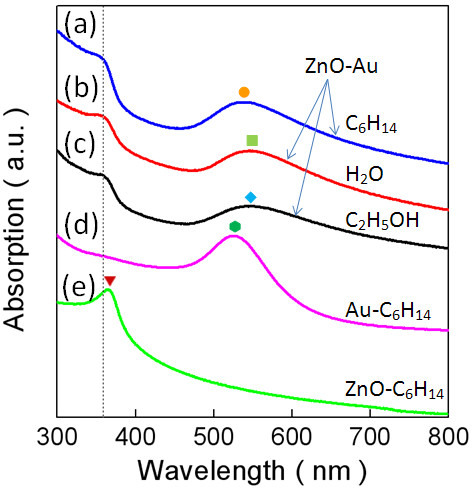
**UV-visible absorbance spectra of the polymer-laced ZnO-Au hybrid nanoparticles dispersed in different solvents.** Hexane **(a)**, water **(b)**, and ethanol **(c)**, in comparison to Au **(d)** and ZnO **(e)** nanoparticles (both in hexane).

The optical properties of the polymer-laced ZnO-Au hybrid nanoparticles were evaluated by UV-visible absorption spectroscopy and photoluminescence (PL) spectrometry. As mentioned above, the nanoparticles can be directly dispersed either in an organic or an aqueous medium without further surface decoration. Figure 4 shows the UV-vis spectra of the ZnO-Au nanoparticles dispersed in hexane (a), water (b), and ethanol (c), together with those of Au (d) and ZnO (e) nanocrystals in similar sizes dispersed in hexane. Clearly, there are two kinds of absorption bands, one from ZnO and the other from the surface plasmon resonance (SPR) of the nanosized Au. In Figure 4a, the ZnO-Au nanoparticles dispersed in hexane exhibit one well-defined absorption band around 356 nm, which is the most distinctive absorption of the ZnO semiconductor [12,32], indicating a blueshift with respect to the absorption peak of the ZnO nanoparticles in hexane at the position of approximately 365 nm, as shown in Figure 4e. In contrast, the effects of solvents on the characteristic absorption band are unambiguously detected in the UV-vis spectra of the polymer-laced ZnO-Au nanoparticles dispersed in water and ethanol. As shown in Figure 4b,c, relative to that in hexane, these are slightly red-shifted to 358 nm for the former and 360 nm for the latter but still definitely blueshifted against the ZnO nanoparticles. The blueshifting of the ZnO absorption may be in principle understood in the quantum confinement due to the reduced particle dimension and the solvent effects [10], as described by the expression

$$\begin{array}{ll} E_{g}\left(R\right)=E_{g}\left(\mathrm{bulk}\right) & +\frac{\hbar^{2}\pi^{2}}{2R^{2}}\left[\frac{1}{m_{e}}+\frac{1}{m_{h}}\right]-\frac{1.8e^{2}}{\epsilon_{2}R}\\ & +\frac{e^{2}}{R}\langle {\overset{\infty }{\underset{m=1}{\sum }}\alpha_{m}\left(\frac{r}{R}\right)}^{2m}\rangle , \end{array}$$

where $\alpha_{m}=\frac{\left(m+1\right)\left(\epsilon -1\right)}{\epsilon_{2}\left(\mathrm{m\epsilon}+m+1\right)}$ and *ϵ* = *ϵ*_2_/*ϵ*_1_.

In the expression, *E*_g_(*R*) and *E*_g_(bulk) represent the bandgap energies of the nanoparticles of radius *R* and the bulk material with a dielectric constant *ϵ*_2_ surrounded in a medium of dielectric constant *ϵ*_1_. The parameters *m*_e_ and *m*_h_ indicate the effective masses of the electron and the hole of the exciton, whereas *e* is the electron charge and *ħ* the Planck constant divided by 2π. The bracket <> means average over a wave function of position *r*. In addition to the change observed in the band positions from the ZnO nanoparticles to the Au-ZnO nanoparticles, comparing the shapes of the bandgap absorption in Figure 4a,e further sheds light on the impact of Au on ZnO, in which the Au-ZnO nanoparticles show increased absorption intensity with the decreasing wavelength against the almost flat absorption of the ZnO nanoparticles. As revealed in the multiple domain nanostructure from the TEM analysis above, moreover, the Au nanocrystallites in the hybrid nanoparticles produce more surface and interface defects, i.e., imperfect lattices and oxygen vacancies that are expected to generate a defect level in the energy band, resulting in likely contributions of more induced excitons and increased exciton density to the moderate enhancement in the absorption intensity in the UV range. Furthermore, the SPR action induced by the Au nanocrystallites, which is to be addressed below, offers additional channels to absorb the incident electromagnetic waves and thus probably augment the UV absorption of the hybrid nanoparticles.

The second well-defined absorption between 520 and 550 nm features the optical property of surface plasmon resonance in consequence of Au nanostructuring [27,28,33,34]. Dependent on the solvent, the peak position of the plasmon band in the solution of the Au-ZnO nanoparticles varies from approximately 533 nm in hexane, approximately 550 nm in water, to approximately 542 nm in ethanol, in comparison to the Au nanoparticles in hexane which has an absorption peaking at approximately 525 nm. Nominally, the peak position and band shape of the plasmon resonance may be subject to factors of composition, dimension, nanostructure shape, dielectric medium, and nanostructuring of the nanoparticle system [33-35]. The distinctly broadening and red-shifting of the surface plasmon spectra of the ZnO-Au hybrid nanoparticles could be due to the fact that the strong interfacial coupling between Au and ZnO results in electron transfer from Au to ZnO taking account of the formation of ZnO-Au nanocomposites [12,33,34]. It is useful to point out that the Au atoms sitting on the surface of the ZnO-Au nanoparticles covered by PEO-PPO-PEO, which is observed as a result of the plasmon resonance addressed above and tested in the experiment, enable thiolation linkage to other molecules [8].

**Figure 5:**
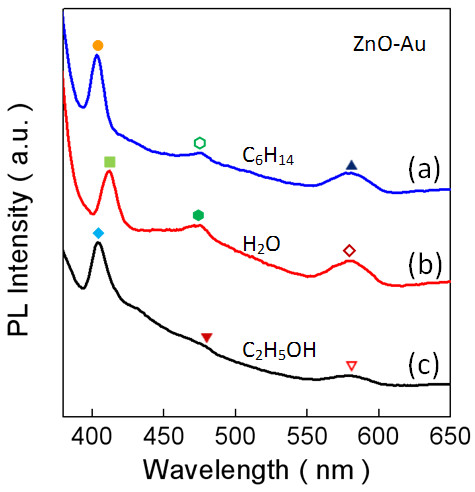
**Photoluminescence emission spectra of the polymer-laced ZnO-Au hybrid nanoparticles dispersed in different solvents.** Hexane **(a)**, water **(b)**, and ethanol **(c)**.

The PL emission spectra of the PEO-PPO-PEO-laced ZnO-Au hybrid nanoparticles respectively dispersed in hexane, water, and ethanol were examined under the excitation wavelength of 360 nm. As shown in Figure 5a, the ZnO-Au nanoparticles in hexane manifest a strong emission peaking at approximately 403 nm, with a weak but firm plateau ending at around 476 nm and a relatively strong emission at approximately 581 nm. In Figure 5b, the nanoparticles in water similarly demonstrate a strong emission at approximately 412 nm, with an analogous, more distinct plateau and a second emission at approximately 580 nm. In the case of ethanol, the nanoparticles show almost the same emission at approximately 404 nm as in hexane, but the plateau becomes nearly indiscernible with the termination at approximately 479 nm and a weaker emission at approximately 578 nm. It is notable that below 400 nm, the spectra show increasing emission with the decreasing wavelength, which could be considered as the enhanced effects of nanosizing of the polymer-laced ZnO-Au nanoparticles. Overall, the blue bands around 400 nm most likely occurs from the donor level of interstitial Zn to the acceptor energy level of Zn vacancy, and the other emission at approximately 580 nm is commonly attributed to the singly ionized oxygen vacancy in ZnO which is due to the recombination between the electrons in a deep defect level or a shallow surface defect level and the holes in a valence band [36]. When nanosized Au combined with ZnO, the electrons accumulate at the interface between Au and ZnO, the electron transfer from Au to ZnO leads to zinc interface defects, and the probability of surface-trapped holes decreases. As a consequence, the electron-hole recombination correspondingly declines, so the visible emissions or defect emissions become weaker and slightly shift [37]. Nonetheless, the contributions of the Au nanocrystallites to the PL emissions may be further understood in two more folds: (1) Referring to the discussion on the absorption above, the presence of the nanocrystallites brings about more surface and interface defects, or more induced excitons and/or increased exciton density, so energetic interactions between the incident electromagnetic waves and the hybrid nanoparticles are boosted to affect the relevant PL emissions, as evidenced, for instance, by the plateau emissions in Figure 5. (2) Mechanistically, the abundant free electrons in the Au nanocrystallites engender the electronic density waves that have their own wavelength depending on the size and shape. Reviewing Figures 4a and 5a, the peak positions of the SPR resonance and the PL emission are comparable. As a result, two opposing mechanisms arise. In one aspect, the electrons in the defect level of ZnO can be excited to the conduction band by the energy transfer via the SPR mode of the Au nanocrystallites activated by the incident electromagnetic waves so that the exciton density increases and consequently, the probability of the relevant emissions is improved. On the other aspect, the emitted photons may be absorbed by the Au nanocrystallites through exciting surface plasmon waves. Such energy dispersion reduces the corresponding PL emission. We remark that many factors can play a decisive role in the quenching and enhancement mechanisms of photoluminescence, and their effects are still in debate. An appropriate elucidation of the mechanisms is of great interest and challenging, which is particularly true for complicated systems such as the present case.

## Conclusions

In summary, we have synthesized the amphiphilic ZnO-Au hybrid nanoparticles by the one-pot non-aqueous nanoemulsion process adopting the biocompatible and non-toxicity triblock copolymer PEO-PPO-PEO as the surfactant. The FTIR assessment substantiates the lacing of the PEO-PPO-PEO macromolecules onto the surface of the nanoparticles. The morphology and structural analyses show the narrow particle size distribution and high crystallinity of the polymer-laced nanoparticles. Moreover, the optical measurements present the well-defined absorption band of the nanoparticles dispersed in different polar and non-polar solvents, manifesting both the ZnO bandgap absorption and the surface plasmon resonance of the nanosized Au, whereas the fluorescent properties reveal multiple fingerprint emissions. Such bi-phase dispersible ZnO-Au nanoparticles could be applicable in biological detection, solar cells, and photocatalysis.

## Competing interests

The authors declare that they have no competing interests.

## Authors’ contributions

XHW, XYZ, and WZC synthesized the nanoparticles and measured the microstructure. HQS, XL and XML measured, and analyzed the optical properties of the nanoparticles. This research work was carried out under the instruction of HLL and JHW. All authors contributed to discussing the results and writing the manuscript. All authors read and approved the final manuscript.
